# Predictive value of controlling nutritional status score for prostate cancer diagnosis

**DOI:** 10.3389/fonc.2024.1268800

**Published:** 2024-02-21

**Authors:** Jiaguo Huang, Ji Sun, Runmiao Hua, Yi Fan, Kai Wang, Liying Zheng, Biao Qian

**Affiliations:** ^1^ Department of Urology, Affiliated Xiaoshan Hospital, Hangzhou Normal University, Hangzhou, China; ^2^ Department of Urology, Affiliated Hangzhou First People’s Hospital, Zhejiang University School of Medicine, Hangzhou, China; ^3^ Department of Graduate, The First Affiliated Hospital of Gannan Medical College, Ganzhou, China; ^4^ Department of Urology, The First Affiliated Hospital of Gannan Medical College, Ganzhou, China

**Keywords:** prostate cancer, controlling nutritional status score, nutritional status, diagnosis, predictive value

## Abstract

**Objective:**

This study aims to explore the predictive value of the Controlling Nutritional Status (CONUT) score for prostate cancer (PCa) diagnosis.

**Methods:**

The data of 114 patients who underwent prostate needle biopsies from June 2020 to December 2022 were retrospectively analyzed. The relationship between CONUT score and various clinical factors as well as PCa diagnosis was evaluated.

**Results:**

The pathological results classified patients into the PCa (n = 38) and non-PCa (n = 76) groups. Compared with the non-PCa group, the PCa group exhibited statistically significant differences in age, prostate-specific antigen (PSA), PSA density (PSAD), the proportion of PI-RADS ≥ 3 in mpMRI, and the CONUT score, prostate volume, lymphocyte count, and total cholesterol concentration (*p* < 0.05). ROC curve analyses indicated the diagnostic accuracy as follows: age (AUC = 0.709), prostate volume (AUC = 0.652), PSA (AUC = 0.689), PSAD (AUC = 0.76), PI-RADS ≥ 3 in mpMRI (AUC = 0.846), and CONUT score (AUC = 0.687). When CONUT score was combined with PSA and PSAD, AUC increased to 0.784. The AUC of CONUT score combined with PSA, PSAD, and mpMRI was 0.881, indicates a higher diagnostic value. Based on the optimal cut-off value of CONUT score, compared with the low CONUT score group, the high CONUT score group has a higher positive rate of PCa diagnosis (*p* < 0.05).

**Conclusion:**

CONUT score is an excellent auxiliary index for PCa diagnosis in addition to the commonly used PSA, PSAD, and mpMRI in clinical practice. Further prospective trials with a larger sample size are warranted to confirm the present study findings.

## 1 Introduction

Prostate cancer (PCa) is a common malignancy diagnosed in men (1). In recent years, the incidence and mortality rates have risen with the increase in life expectancy and the change in dietary structure and medical treatment (1). According to the Global Cancer Statistics from the United States, PCa accounted for 7.3% of all new male cancer cases worldwide in 2020 (2). However, the incidence rate of PCa in China is rapidly increasing, and it is now the most common malignancy of the male genitourinary system (3).

A favorable prognosis for PCa depends on an early diagnosis, which remains challenging. PCa has no apparent symptoms in early stage, making early diagnosis difficult. PCa diagnosis is based on the histopathological evaluation of a prostate needle biopsy. The prostate-specific antigen (PSA) is a specific tumor biomarker of the prostate that plays a crucial role in the diagnosis of early occult PCa. PSA screening has increased the detection rate of PCa. However, in the subsequent promotion and application of PSA screening, many screening trials have revealed its shortcomings, including low survival benefits, excessive diagnosis and waste of medical resources, and varying degrees of pressure on society and families (4–6). The prostate needle biopsy is an invasive procedure that may cause pain, infection, bleeding, and psychological burden. Recently, some PSA-related test parameters have been employed to improve the accuracy of PCa prediction. These parameters include the free/total PSA ratio, PSA density (PSAD), PSA doubling time (PSAdt), and prostate health index (PHI) (7). Concurrently, some interesting methodologies for predicting PCa risk have emerged, such as employing machine learning approach for PCa classification based on clinical biomarkers (8). However, despite these advancements, overdiagnosis and overtreatment are found. Therefore, new biomarkers are necessary to help judge whether to perform a biopsy and reduce unnecessary prostate needle biopsies in low-risk cancer patients.

Malnutrition is one of the most common complications of cancer patients, and it can even lead to severe consequences of cachexia (9, 10), which may be caused by tumor-related anorexia, inflammation, and metabolic changes. Age, disease stage, and tumor type affect the degree of malnutrition. Malnutrition has several harmful effects on cancer patients, including weight loss and reduced survival rates (11, 12). Immuno-nutritional evaluation among oncological patients is an interesting and ongoing topic of debate. Nutritional status can predict the prognosis of PCa, urothelial carcinoma and renal cell carcinoma (13–17). The current study is mainly used in the judgment of disease prognosis, including complications and mortality rate. For example, malnourished patients have higher complications and mortality who treated with radical cystectomy. Zhang et al. proposed that the Controlling Nutritional Status (CONUT) score is a new prognostic indicator of PCa prognosis (17). A study investigated 1317 patients with malignancy, and below adequate body mass index (BMI) was observed through anthropometric analysis in 10.85% of the patients at the time of diagnosis (18). To the authors’ knowledge, however, no study has reported the predictive value of nutritional status in PCa diagnosis. Currently, serum albumin and BMI are primarily used to evaluate the nutritional status of patients, whereas patients with early-stage malignant tumors often exhibit no apparent changes. The CONUT score—an index of nutritional status—is calculated based on patients’ lymphocytes count, serum albumin concentration, and total cholesterol concentration (TCH) (19). A prognostic role of the CONUT score for prediction of OS, CSS, and RFS in cancer patients was shown by 91.7%, 90.9%, and 52.6% of the studies, respectively (20). Whether the CONUT score is a reliable predictor of PCa diagnosis is a question of interest for investigation. The present study analyzed the clinical data of patients who underwent prostate needle biopsies and explored the predictive value of the CONUT score in PCa diagnosis.

## 2 Patients and methods

### 2.1 Patient characteristics

Patients who underwent prostate needle biopsies in the urology department of Xiaoshan Hospital in Zhejiang Province between June 2020 and December 2022 constituted the study subjects. For the indications of prostate needle biopsy, refer to the Chinese Urological Disease Diagnosis and Treatment Guide, and exclude patients with symptomatic prostatitis, renal failure, liver failure, active infection, rheumatism, or other malignancy. This study was approved by the Ethics Committee of Zhejiang Xiaoshan Hospital (ID number: 2021-010, Date: January 19, 2021).

### 2.2 Clinical and laboratory assessments

The CONUT score was calculated using the preoperative lymphocyte count, serum albumin concentration, and TCH (**Table 1**) as follows: CONUT score = lymphocyte count score + serum albumin concentration score + TCH score. The general data of patients—such as age, body mass index (BMI), smoking and alcohol history, PSA, prostate volume (PV), PSAD, and multiparametric magnetic resonance imaging (mpMRI)—and pathological results, including the Gleason score of PCa patients, were also collected. PSA density (PSAD) was calculated by dividing the PSA level by the PV. All the laboratory examinations were blood drawn at the same time.

**Table 1:** Assessment of nutrition status by the CONUT score.

Parameters	None	Light	Moderate	Severe
Lymphocyte count (10^9^/L)	≥1.600	1.200-1.599	0.800-1.199	<0.800
Score	0	1	2	3
Serum albumin concentration (g/dL)	≥35	30-34.9	25-29	<29
Score	0	2	4	6
Total cholesterol concentration (mg/dL)	≥180	140-179	100-139	<100
Score	0	1	2	3

CONUT, Controlling Nutritional Status.

### 2.3 Statistical analysis

Data analysis was performed using SPSS 23.0 and MedCalc Software. The Student’s t-test was used for normally distributed parameters, whereas the Wilcoxon rank-sum test was used for non-normally distributed parameters. Continuous variables are expressed as mean ± standard deviation. Qualitative variables are presented as numbers (n) and percentages (%). Chi-square tests were used to compare qualitative variables. Receiver operating characteristic (ROC) curves were plotted and corresponding 95% CIs, area under the curve (AUC), sensitivity, specificity, and optimal cutoff calculated. The optimal cut-off value was determined using the Youden’s index (Youden’s index = sensitivity + specificity - 1), and the AUC represented the diagnostic performance. A larger AUC indicates a higher diagnostic value. And logistic regression analysis was adopted to derive combined diagnostic factors to conduct ROC combined diagnosis. *p* < 0.05 was considered significant.

## 3 Results

A total of 114 patients were enrolled in the study. **Table 2** shows the basic characteristics of all patients included in the study. The average age was 68.93 ± 9.05 years old, the average BMI was 23.58 ± 3.01 kg/m^2^, the median PV was 40.31 cm^3^, the median PSA was 10.02 ng/mL, and the median PSAD was 0.25 ng/mL × cm^3^. In the report of mpMRI, 15.79%, 37.72%, 30.7%, 14.04%, and 1.75% of patients had PI-RADS 1, PI-RADS 2, PI-RADS 3, PI-RADS 4, and PI-RADS 5, respectively. According to the pathological diagnosis of prostate needle biopsies, there were 76 patients (66.67%) with non-PCa and 38 patients (33.33%) with PCa. Based on the Gleason score classification during 38 PCa patients, 8 patients had a tumor with a Gleason score of 6 (21.05%), 19 patients presented a Gleason score of 7 (50%), 6 patients presented a Gleason score of 8 (15.79%), 4 patients presented a Gleason score of 9 (10.53%), and 1 patient presented a Gleason score of 10 (2.63%). There were 26 patients (29.55%), 21 patients (23.86%), 20 patients (22.73%), 12 patients (13.64%), 5 patients (5.68%), 1 patient (1.14%), and 3 patients (3.41%) with respective CONUT scores of 0, 1, 2, 3, 4, 5, and 6, respectively.

**Table 2:** Basic characteristics of patients.

Parameters	Value or number of patients
Age (years old)	68.93 ± 9.05
BMI (kg/m^2^)	23.58 ± 3.01
Prostate volume (cm^3^)	40.31
PSA (ng/mL)	10.02
PSAD (ng/mL × cm^3^)	0.25
mpMRI	
PI-RADS 1	18 (15.79%)
PI-RADS 2	43 (37.72%)
PI-RADS 3	35 (30.7%)
PI-RADS 4	16 (14.04%)
PI-RADS 5	2 (1.75%)
Prostate cancer	38 (33.33%)
Gleason score	
6	8 (21.05%)
7	19 (50%)
8	6 (15.79%)
9	4 (10.53%)
10	1 (2.63%)
CONUT score	
0	17 (14.91%)
1	29 (25.44%)
2	35 (30.7%)
3	24 (21.05%)
4	8 (7.02%)
5	1 (0.88%)

BMI, body mass index; PSA, prostate-specific antigen; PSAD, PSA density; mpMRI, multiparametric magnetic resonance imaging; CONUT, Controlling Nutritional Status.

The pathological results classified patients into the PCa (n = 38) and non-PCa (n = 76) groups. **Table 3** presents the main characteristics of the PCa and non-PCa groups. The PCa group exhibited statistically significant differences in age (*p* = 0.001), PSA (*p* = 0.001), PSAD (*p* < 0.001), the proportion of PI-RADS ≥ 3 in mpMRI (*p* < 0.001), and CONUT score (*p* = 0.001), PV (*p* = 0.008), lymphocyte count (*p* = 0.016) and TCH (*p* < 0.001) compared to the non-PCa group; however, there was no statistical difference in BMI, albumin concentration, smoking history and alcohol history.

**Table 3:** Comparison of basic clinical data between PCa and non-PCa groups.

Parameters	PCa group (n=38)	non-PCa group (n=76)	*p* value
Age (years old)	72.95 ± 7.00	66.92 ± 9.32	0.001
BMI (kg/m^2^)	23.84 ± 3.52	23.45 ± 2.75	0.512
Smoking history	9	28	0.157
Alcohol history	9	21	0.652
Prostate volume (cm^3^)	32.28	42.83	0.008
PSA (ng/mL)	12.22	8.77	0.001
PSAD (ng/mL × cm^3^)	0.42	0.22	<0.001
mpMRI (PI-RADS ≥ 3)	32	21	<0.001
Lymphocyte count (10^9^/L)	1.22 ± 0.35	1.43 ± 0.46	0.016
Serum albumin concentration (g/dL)	43.18 ± 3.75	43.45 ± 3.15	0.695
Total cholesterol concentration (mg/dL)	163.95 ± 34.97	187.48 ± 31.36	<0.001
CONUT score	2	1.5	0.001

PCa, prostate cancer; BMI, body mass index; PSA, prostate-specific antigen; PSAD, PSA density; mpMRI, multiparametric magnetic resonance imaging; CONUT, Controlling Nutritional Status.

**Figure 1:**
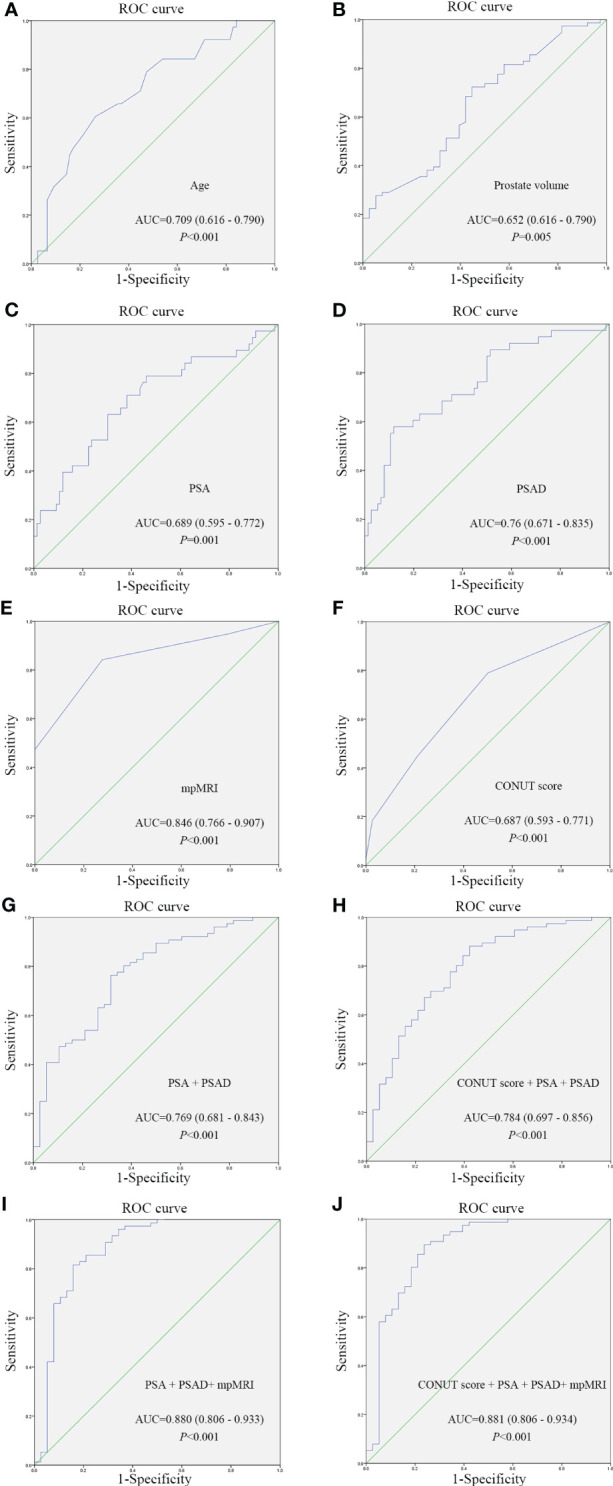
ROC curves of different parameters for assessing the predictive value of different parameters for prostate cancer diagnosis. **(A)** ROC curve of Age; **(B)** ROC curve of Prostate volume; **(C)** ROC curve of PSA; **(D)** ROC curve of PSAD; **(E)** ROC curve of mpMRI; **(F)** ROC curve of CONUT score; **(G)** Combined diagnosis ROC curve of PSA and PSAD; **(H)** Combined diagnosis ROC curve of CONUT score, PSA and PSADand; **(I)** Combined diagnosis ROC curve of PSA, PSAD and mpMRI; **(J)** Combined diagnosis ROC curve of CONUT score, PSA, PSAD and mpMRI. CONUT, Controlling Nutritional Status; PSA, prostate-specific antigen; PSAD, PSA density; mpMRI, multiparametric magnetic resonance imaging; ROC, receiver operating characteristic.

Based on patient characteristics, the present study investigated the diagnostic value of these aforementioned parameters for PCa and compared them with PSA. Diagnostic accuracy was evaluated by the area under the curve (AUC) from ROC curves (**Table 4**), the AUCs of these parameters were as follows: age, 0.709 (95% confidence interval [CI]: 0.616-0.790) (**Figure 1A**); PV, 0.652 (95% CI: 0.557-0.738) (**Figure 1B**); PSA, 0.689 (95% CI: 0.595-0.772) (**Figure 1C**); PSAD, 0.76 (95% CI: 0.671-0.835) (**Figure 1D**); mpMRI (PI-RADS ≥ 3), 0.846 (95% CI: 0.766-0.907) (**Figure 1E**); CONUT score, 0.687 (95% CI: 0.593-0.771) (**Figure 1F**). Among the parameters, mpMRI (PI-RADS ≥ 3) had the highest AUC value. However, when the CONUT score was combined with PSA and PSAD, the AUC ascended to 0.784 (95% CI: 0.806 - 0.934) (**Figure 1H**) was higher than without the CONUT score (0.769, 95% CI: 0.681-0.843) (**Figure 1G**). An evaluation of the diagnostic value of CONUT score, PSA, PSAD, and mpMRI (PI-RADS ≥ 3) yielded an AUC of 0.881 (95% CI: 0.806-0.934) and a specificity of 89.47% (**Figure 1J**), which was higher than without the CONUT score (0.880, 95% CI: 0.806-0.933) (**Figure 1I**), indicates a higher diagnostic value. Therefore, it can be inferred that the CONUT score serves as a valuable supplementary indicator that enhances the diagnostic accuracy for PCa diagnosis.

**Table 4:** AUC were used to assess the diagnostic value of different parameters for PCa.

Parameters	AUC	95% CI	*p* value	Optimal cut-off value	Sensitivity (%)	Specificity (%)
Age	0.709	0.616 - 0.790	<0.001	71	60.53	73.68
Prostate volume	0.652	0.557 - 0.738	0.005	32.72	55.26	72.37
PSA	0.689	0.595 - 0.772	0.001	9.21	78.95	53.95
PSAD	0.76	0.671 - 0.835	<0.001	0.38	57.89	88.16
mpMRI	0.846	0.766 - 0.907	<0.001	2	84.21	72.37
PSA + PSAD	0.769	0.681-0.843	<0.001	0.45	68.42	76.32
PSA + PSAD + mpMRI	0.880	0.806-0.933	<0.001	0.66	84.21	81.58
CONUT score	0.687	0.593 - 0.771	<0.001	1	78.95	50.00
PSA + PSAD	0.769	0.681 - 0.843	<0.001	0.71	68.42	76.32
CONUT score + PSA + PSAD	0.784	0.697 - 0.856	<0.001	0.37	57.89	88.16
PSA + PSAD + mpMRI	0.880	0.806 - 0.933	<0.001	0.61	84.21	81.58
CONUT score + PSA + PSAD + mpMRI	0.881	0.806 - 0.934	<0.001	0.26	76.32	89.47

AUC, area under the curve; PCa, prostate cancer; CI, confidence interval; PSA, prostate-specific antigen; PSAD, PSA density; mpMRI, multiparametric magnetic resonance imaging; CONUT, Controlling Nutritional Status.

According to ROC curve analysis, the optimal cut-off value of the CONUT score for PCa was 1, and the specificity and sensitivity were 78.95% and 50%, respectively (**Table 4**, **Figure 1F**). According to the cut-off value of the CONUT score (CONUT score = 1), 114 patients who underwent prostate needle biopsies were divided into low (≤ 1) and high (>1) CONUT score groups. **Table 5** shows a significant difference in age, PSA, PSAD, and positive rate of PCa (*p* < 0.05). The high CONUT score group has a higher age, PSA, PSAD, and positive rate of PCa diagnosis. Regarding other clinical characteristics, there was no statistical difference between the high and low CONUT score groups.

**Table 5:** Comparison of basic clinical data between low (≤ 1) and high (>1) CONUT score groups.

Parameters	CONUT score		*p* value
	Low (n=46)	High (n=68)	
Age (years old)	64.39 ± 8.87	72 ± 7.84	<0.001
BMI (kg/m^2^)	24.17 ± 2.64	23.19 ± 3.21	0.089
Smoking history	15/46	22/68	0.977
Alcohol history	11/46	19/68	0.632
Prostate volume (cm^3^)	40.53	40.05	0.933
PSA (ng/mL)	8.63	11.25	0.02
PSAD (ng/mL × cm^3^)	0.23	0.28	0.042
mpMRI (PI-RADS ≥ 3)	18/46	35/68	0.195
Positive rate of PCa	9/46	22/68	0.03
Gleason score	7.11 ± 0.99	7.45 ± 1.03	0.254

CONUT, Controlling Nutritional Status; BMI, body mass index; PSA, prostate-specific antigen; PSAD, PSA density; mpMRI, multiparametric magnetic resonance imaging; PCa, prostate cancer.

## 4 Discussion

In this study, the clinical value of the CONUT score in PCa diagnosis was evaluated for the first time. The CONUT score of the PCa group was higher than that of the non-PCa group, whereas the difference in serum albumin and BMI between the two groups was not statistically significant. Secondly, the high CONUT score group was likelier to report PCa through biopsy than the low CONUT score group. The present study also confirmed that PSA remains an important diagnostic marker of PCa and has high diagnostic value. Furthermore, PSAD and mpMRI provide strong support for the diagnosis of PCa. The diagnostic value is higher when the CONUT score is combined with PSA and PSAD, or when the CONUT score is combined with PSA, PSAD, and mpMRI. Based on these findings, the present study concluded that the CONUT score may be a valuable auxiliary index for PCa diagnosis in addition to the PSA, PSAD, and mpMRI currently used in clinics.

The prevalence of malnutrition among cancer patients is between 20% and 70% worldwide (21). Cancer cachexia affects 50%-80% of cancer patients, and 20%-70% of cancer patients have skeletal muscle dystrophy (12) Age, disease stage, and tumor type can affect the risk of malnutrition (22, 23). Serum albumin level and BMI are used to determine a patient’s nutritional status. The present study revealed no statistical difference in serum albumin and BMI between the PCa and non-PCa groups. Serum albumin and BMI alone are insufficient for determining the nutritional status of PCa patients. This finding is mainly because the early stage of cancer has minimal effects on the nutritional status of patients, which is difficult to detect using serum albumin and BMI. Therefore, the present study attempted to better determine the potential risk of malignant tumors based on blood test indicators. This study also attempted to explore the clinical value of the CONUT score for PCa diagnosis because the three evaluation indicators that comprise the CONUT score are generally used and easily detected in clinical work; the CONUT score is used as an indicator for nutritional status evaluation.

The CONUT score is a practical and objective method of evaluating a patient’s nutritional status. The serum albumin concentration is a reliable indicator to judge the nutritional status and immune-inflammatory reaction of cancer patients and is closely associated with their survival rate (24, 25). Many inflammatory factors, such as cytokines and c-reactive protein, are produced by tumor-related inflammatory reactions. These inflammatory factors can regulate albumin synthesis (26). Furthermore, studies have found that hypoalbuminemia is related to immune injury and tolerance, promoting tumor cell proliferation and disease progression (27). In this study, although the serum albumin concentration of non-PCa patients is lower than that of PCa patients, there is no statistical significance, confirming that it is challenging to demonstrate changes in serum albumin and BMI because the early stage of cancer has little effect on the nutritional status of patients.

Many studies have confirmed the value of inflammatory indicators, such as monocyte-to-lymphocyte and platelet-to-lymphocyte ratios, for PCa diagnosis (28–30). Lymphocytes play an essential role in the immune response to cancer because they induce cell apoptosis, inhibit tumor growth and metastasis, and mediate cytotoxicity. Furthermore, studies have demonstrated that a decline in lymphocyte counts indicates a decrease in immune function and that the immune system cannot respond adequately to cancer cells. The formation of microenvironments facilitates cancer cell proliferation and metastasis, leading to poorer clinical outcomes (31, 32). In this study, the lymphocyte count of the non-PCa group is lower than that of the PCa group, which is consistent with the findings of previous studies.

TCH is a component of the CONUT scoring system, which differs from other nutritional status scoring systems. As an essential component of the cell membrane, cholesterol may be related to the proliferation, metastasis, and immune response of tumor cells (33, 34). Muldoon et al. found that patients with hypocholesterolemia had fewer lymphocytes, T lymphocytes, and CD8 + T lymphocytes than patients with hypercholesterolemia (32).

The present study confirms that PSA is still the most important biomarker for PCa diagnosis (35). PSA has disadvantages because it is a prostate-specific antigen but not PCa-specific. In cases of acute prostatitis or benign prostatic hyperplasia, serum PSA levels can rise, making it difficult for PCa diagnosis (36, 37). Additionally, its overuse leads to unnecessary biopsies and related complications. The clinical application of prostate mpMRI and the prostate imaging reporting and data system (PI-RADS) has also improved the diagnosis of PCa and clinically significant PCa in terms of imaging (38). The present study found that the CONUT score combined with PSA and PSAD has a high specificity for PCa diagnosis (0.784). In the medical structure where mpMRI examination is available, the CONUT score combined with PSA, PSAD, and mpMRI has higher specificity in diagnosing PCa (0.881). Even in a medical structure without mpMRI, CONUT score clearly improves the diagnostic value of the combined indicators of PSA and PSAD. The current study has not reported the combination of the aforementioned indicators. We find that AUC from the combination of the aforementioned indicators with CONUT score was higher than without CONUT score. From these data, the present study conclude that the CONUT score may be an excellent auxiliary index for PCa diagnosis in addition to PSA, PSAD, and mpMRI commonly used in clinical practice. These results also demonstrated that the cut-off value of the CONUT score of 1 was significant, and patients with a high CONUT score who reported PCa by biopsy were higher than those with a low CONUT score. Therefore, the present study assume that the significance of the CONUT score for PCa diagnosis would help clinicians identify high-risk patients in time and decide whether to conduct further prostate biopsies. Combining the CONUT score with PSA may help compensate for a deficiency in PSA screening.

Since the three evaluation indexes of CONUT score are commonly used and easy to detect in clinical practice, the CONUT score has the advantage of being simple, cost-effective and reliable in predicting PCa diagnosis, thereby it may also reduce the number of unnecessary clinical biopsies. It is hope that more similar studies can be reported. However, the results of this study should be interpreted in the context of several limitations. Firstly, this study was a retrospective study from a single center, which limited its power and generalizability. Secondly, the sample size was relatively limited, which made it difficult to conduct further verification analysis by dividing patients based on various PSA ranges. Therefore the present study findings would be more meaningful to be confirmed with further prospective research with a larger sample size including various PSA ranges.

## 5 Conclusion

The CONUT score is an excellent auxiliary index for PCa diagnosis in addition to the commonly used PSA, PSAD, and mpMRI in clinical practice. The evaluation significance of the CONUT score for PCa will help clinicians identify high-risk patients in time and decide whether to conduct a further prostate biopsy. The combined use of the CONUT score and PSA may help reduce the number of unnecessary clinical biopsies. Further prospective trails with a larger sample size are warranted to confirm the present study findings because this study is a retrospective study with a limited sample size.

## Data availability statement

The original contributions presented in the study are included in the article/supplementary material. Further inquiries can be directed to the corresponding author.

## Ethics statement

The studies involving humans were approved by the ethical committee of Affiliated Xiaoshan Hospital, Hangzhou Normal University. The studies were conducted in accordance with the local legislation and institutional requirements. The ethics committee/institutional review board waived the requirement of written informed consent for participation from the participants or the participants’ legal guardians/next of kin because the study was a retrospective study.

## Author contributions

JH: Formal analysis, Funding acquisition, Writing – original draft. JS: Writing – original draft, Data curation, Investigation. RH: Data curation, Writing – review & editing. YF: Supervision, Writing – review & editing. KW: Funding acquisition, Writing – review & editing. LZ: Writing – review & editing. BQ: Conceptualization, Methodology, Supervision, Writing – review & editing.

## References

[1] Kimura T Sato S Takahashi H Egawa S. Global trends of latent prostate cancer in autopsy studies. Cancers (Basel) (2021) 13. doi: 10.3390/cancers13020359 PMC7835858 33478075

[2] Sung H Ferlay J Siegel RL Laversanne M Soerjomataram I Jemal A. Global cancer statistics 2020: GLOBOCAN estimates of incidence and mortality worldwide for 36 cancers in 185 countries. CA Cancer J Clin (2021) 71:209–49. doi: 10.3322/caac.21660 33538338

[3] Xing L Xiaoyong Z. Progress in the epidemiology of prostate cancer in China. Cancer Res Prev Treat (2021) 48:98–102.

[4] Pinsky PF Black A Daugherty SE Hoover R Berndt SI. Metastatic prostate cancer at diagnosis and through progression in the prostate, lung, colorectal, and ovarian cancer screening trial. Cancer (2019) 125:2965–74. doi: 10.1002/cncr.32176 PMC6690759 31067347

[5] Hugosson J Roobol MJ Månsson M Tammela T Zappa M Nelen V. A 16-yr follow-up of the European randomized study of screening for prostate cancer. Eur Urol (2019) 76:43–51. doi: 10.1016/j.eururo.2019.02.009 30824296 PMC7513694

[6] Auvinen A Moss SM Tammela TL Taari K Roobol MJ Schröder FH. Absolute effect of prostate cancer screening: balance of benefits and harms by center within the European randomized study of prostate cancer screening. Clin Cancer Res (2016) 22:243–9. doi: 10.1158/1078-0432.CCR-15-0941 PMC4951205 26289069

[7] Mottet N van den Bergh R Briers E Van den Broeck T Cumberbatch MG De Santis M. EAU-EANM-ESTRO-ESUR-SIOG guidelines on prostate cancer-2020 update. Part 1: screening, diagnosis, and local treatment with curative intent. Eur Urol (2021) 79:243–62. doi: 10.1016/j.eururo.2020.09.042 33172724

[8] Özhan O Yağın FH. Machine learning approach for classification of prostate cancer based on clinical biomarkers. J Cogn Sys (2022) 7:17–20. doi: 10.52876/jcs.1221425

[9] Agarwal E Ferguson M Banks M Bauer J Capra S Isenring E. Nutritional status and dietary intake of acute care patients: results from the Nutrition Care Day Survey 2010. Clin Nutr (2012) 31:41–7. doi: 10.1016/j.clnu.2011.08.002 21862187

[10] Marshall KM Loeliger J Nolte L Kelaart A Kiss NK. Prevalence of malnutrition and impact on clinical outcomes in cancer services: A comparison of two time points. Clin Nutr (2019) 38:644–51. doi: 10.1016/j.clnu.2018.04.007 29789167

[11] Van Cutsem E Arends J. The causes and consequences of cancer-associated malnutrition. Eur J Oncol Nurs (2005) 9:S51–63. doi: 10.1016/j.ejon.2005.09.007 16437758

[12] Ryan AM Power DG Daly L Cushen SJ Ní BĒ Prado CM. Cancer-associated malnutrition, cachexia and sarcopenia: the skeleton in the hospital closet 40 years later. Proc Nutr Soc (2016) 75:199–211. doi: 10.1017/S002966511500419X 26786393

[13] Ornaghi PI Afferi L Antonelli A Cerruto MA Odorizzi K Gozzo A. The impact of preoperative nutritional status on post-surgical complication and mortality rates in patients undergoing radical cystectomy for bladder cancer: a systematic review of the literature. World J Urol (2021) 39:1045–81. doi: 10.1007/s00345-020-03291-z 32519225

[14] Munbauhal G Drouin SJ Mozer P Colin P Phé V Cussenot O. Malnourishment in bladder cancer and the role of immunonutrition at the time of cystectomy: an overview for urologists. BJU Int (2014) 114:177–84. doi: 10.1111/bju.12529 24410904

[15] Claps F Mir MC van Rhijn B Mazzon G Soria F D’Andrea D. Impact of the controlling nutritional status (CONUT) score on perioperative morbidity and oncological outcomes in patients with bladder cancer treated with radical cystectomy. Urol Oncol (2023) 41:49.e13–22. doi: 10.1016/j.urolonc.2022.09.023 36274030

[16] Takemura K Yuasa T Fujiwara R Ito M Suzuki H Yonese J. Prognostic significance of the controlling nutritional status (CONUT) score in patients with advanced renal cell carcinoma treated with nivolumab after failure of prior tyrosine kinase inhibitors. J Urol (2020) 204:1166–72. doi: 10.1097/JU.0000000000001196 32567459

[17] Zhang W Wu Y Zhang Z Guo Y Wang R Wang L. Controlling Nutritional Status score: A new prognostic indicator for patients with oligometastatic prostate cancer. Curr Probl Cancer (2019) 43:461–70. doi: 10.1016/j.currproblcancer.2019.02.001 30910226

[18] Lemos Pdos S de Oliveira FL Caran EM. Nutritional status of children and adolescents at diagnosis of hematological and solid Malignancies. Rev Bras Hematol Hemoter (2014) 36:420–3. doi: 10.1016/j.bjhh.2014.06.001 PMC4318456 25453652

[19] Ignacio de Ulíbarri J González-Madroño A de Villar NG González P González B Mancha A. CONUT: a tool for controlling nutritional status. First validation in a hospital population. Nutr Hosp (2005) 20:38–45. 15762418

[20] Kheirouri S Alizadeh M. Prognostic potential of the preoperative controlling nutritional status (CONUT) score in predicting survival of patients with cancer: A systematic review. Adv Nutr (2021) 12:234–50. doi: 10.1093/advances/nmaa102 PMC7850023 32910812

[21] Arends J Baracos V Bertz H Bozzetti F Calder PC Deutz N. ESPEN expert group recommendations for action against cancer-related malnutrition. Clin Nutr (2017) 36:1187–96. doi: 10.1016/j.clnu.2017.06.017 28689670

[22] Bozzetti F. Screening the nutritional status in oncology: a preliminary report on 1,000 outpatients. Support Care Cancer (2009) 17:279–84. doi: 10.1007/s00520-008-0476-3 18581148

[23] Hébuterne X Lemarié E Michallet M de Montreuil CB Schneider SM Goldwasser F. Prevalence of malnutrition and current use of nutrition support in patients with cancer. JPEN J Parenter Enteral Nutr (2014) 38:196–204. doi: 10.1177/0148607113502674 24748626

[24] Ayhan A Günakan E Alyazıcı İ Haberal N Altundağ Ö Dursun P. The preoperative albumin level is an independent prognostic factor for optimally debulked epithelial ovarian cancer. Arch Gynecol Obstet (2017) 296:989–95. doi: 10.1007/s00404-017-4511-9 28875365

[25] McMillan DC Elahi MM Sattar N Angerson WJ Johnstone J McArdle CS. Measurement of the systemic inflammatory response predicts cancer-specific and non-cancer survival in patients with cancer. Nutr Cancer (2001) 41:64–9. doi: 10.1080/01635581.2001.9680613 12094630

[26] Yeun JY Kaysen GA. Factors influencing serum albumin in dialysis patients. Am J Kidney Dis (1998) 32:S118–25. doi: 10.1016/s0272-6386(98)70174-x 9892378

[27] Cengiz O Kocer B Sürmeli S Santicky MJ Soran A. Are pretreatment serum albumin and cholesterol levels prognostic tools in patients with colorectal carcinoma. Med Sci Monit (2006) 12:CR240–7. 16733481

[28] Xu Z Zhang J Zhong Y Mai Y Huang D Wei W. Predictive value of the monocyte-to-lymphocyte ratio in the diagnosis of prostate cancer. Med (Baltimore) (2021) 100:e27244. doi: 10.1097/MD.0000000000027244 PMC8462614 34559125

[29] Kaynar M Yildirim ME Gul M Kilic O Ceylan K Goktas S. Benign prostatic hyperplasia and prostate cancer differentiation via platelet to lymphocyte ratio. Cancer Biomark (2015) 15:317–23. doi: 10.3233/CBM-150458 PMC12964681 25586096

[30] Caglayan V Onen E Avci S Sambel M Kilic M Oner S. Lymphocyte-to-monocyte ratio is a valuable marker to predict prostate cancer in patients with prostate specific antigen between 4 and 10 ng/dl. Arch Ital Urol Androl (2019) 90:270–5. doi: 10.4081/aiua.2018.4.270 30655640

[31] Orhan A Vogelsang RP Andersen MB Madsen MT Hölmich ER Raskov H. The prognostic value of tumour-infiltrating lymphocytes in pancreatic cancer: a systematic review and meta-analysis. Eur J Cancer (2020) 132:71–84. doi: 10.1016/j.ejca.2020.03.013 32334338

[32] Ray-Coquard I Cropet C Van Glabbeke M Sebban C Le Cesne A Judson I. Lymphopenia as a prognostic factor for overall survival in advanced carcinomas, sarcomas, and lymphomas. Cancer Res (2009) 69:5383–91. doi: 10.1158/0008-5472.CAN-08-3845 PMC2775079 19549917

[33] Kark JD Smith AH Hames CG. Serum retinol and the inverse relationship between serum cholesterol and cancer. Br Med J (Clin Res Ed) (1982) 284:152–4. doi: 10.1136/bmj.284.6310.152 PMC1495538 6799076

[34] Williams RR Sorlie PD Feinleib M McNamara PM Kannel WB Dawber TR. Cancer incidence by levels of cholesterol. JAMA (1981) 245:247–52. doi: 10.1001/jama.1981.03310280023021 7452849

[35] Lippi G Montagnana M Guidi GC Plebani M. Prostate-specific antigen-based screening for prostate cancer in the third millennium: useful or hype. Ann Med (2009) 41:480–9. doi: 10.1080/07853890903156468 19657768

[36] Erol B Gulpinar MT Bozdogan G Ozkanli S Onem K Mungan G. The cutoff level of free/total prostate specific antigen (f/t PSA) ratios in the diagnosis of prostate cancer: a validation study on a Turkish patient population in different age categories. Kaohsiung J Med Sci (2014) 30:545–50. doi: 10.1016/j.kjms.2014.03.008 PMC11916534 25458043

[37] Polascik TJ Oesterling JE Partin AW. Prostate specific antigen: a decade of discovery–what we have learned and where we are going. J Urol (1999) 162:293–306. doi: 10.1016/s0022-5347(05)68543-6 10411025

[38] Mendhiratta N Rosenkrantz AB Meng X Wysock JS Fenstermaker M Huang R. Magnetic resonance imaging-ultrasound fusion targeted prostate biopsy in a consecutive cohort of men with no previous biopsy: reduction of over detection through improved risk stratification. J Urol (2015) 194:1601–6. doi: 10.1016/j.juro.2015.06.078 26100327

